# Nitric oxide synthase mediates cerebellar dysfunction in mice exposed to repetitive blast-induced mild traumatic brain injury

**DOI:** 10.1038/s41598-020-66113-7

**Published:** 2020-06-10

**Authors:** Aric F. Logsdon, Abigail G. Schindler, James S. Meabon, Mayumi Yagi, Melanie J. Herbert, William A. Banks, Murray A. Raskind, Desiree A. Marshall, C. Dirk Keene, Daniel P. Perl, Elaine R. Peskind, David G. Cook

**Affiliations:** 1 Geriatric Research Education and Clinical Center (GRECC), Veterans Affairs Puget Sound Health Care System, Seattle, WA 98108 USA; 2 Division of Gerontology and Geriatric Medicine, Department of Medicine, University of Washington School of Medicine, Seattle, WA 98195 USA; 3 VA Northwest Mental Illness Research Education and Clinical Center, Veterans Affairs Puget Sound Health Care System, Seattle, WA 98108 USA; 4 Department of Psychiatry and Behavioral Sciences, University of Washington School of Medicine, Seattle, WA 98195 USA; 5 Department of Pathology, University of Washington, Seattle, WA 98195 USA; 6 Department of Pathology, Center for Neuroscience and Regenerative Medicine, School of Medicine, Uniformed Services University, Bethesda, MD 20814 USA

**Keywords:** Neuroscience, Blood-brain barrier, Diseases of the nervous system, Motor control, Neuroimmunology, Somatosensory system, Diseases, Trauma, Neurology, Brain injuries

## Abstract

We investigated the role of nitric oxide synthase (NOS) in mediating blood-brain barrier (BBB) disruption and peripheral immune cell infiltration in the cerebellum following blast exposure. Repetitive, but not single blast exposure, induced delayed-onset BBB disruption (72 hours post-blast) in cerebellum. The NOS inhibitor N(G)-nitro-L-arginine methyl ester (L-NAME) administered after blast blocked BBB disruption and prevented CD4^+^ T-cell infiltration into cerebellum. L-NAME also blocked blast-induced increases in intercellular adhesion molecule-1 (ICAM-1), a molecule that plays a critical role in regulating blood-to-brain immune cell trafficking. Blocking NOS-mediated BBB dysfunction during this acute/subacute post-blast interval (24–71 hours after the last blast) also prevented sensorimotor impairment on a rotarod task 30 days later, long after L-NAME cleared the body. In postmortem brains from Veterans/military Servicemembers with blast-related TBI, we found marked Purkinje cell dendritic arbor structural abnormalities, which were comparable to neuropathologic findings in the blast-exposed mice. Taken collectively, these results indicate that blast provokes delayed-onset of NOS-dependent pathogenic cascades that can later emerge as behavioral dysfunction. These results also further implicate the cerebellum as a brain region vulnerable to blast-induced mTBI.

## Introduction

Blast-induced mild traumatic brain injury (mTBI) is prevalent among military personnel and Veterans who have served in Operation Enduring Freedom/Operation Iraqi Freedom/Operation New Dawn (OEF/OIF/OND). Between 2000–2018, more than 380,000 OEF/OIF/OND Veterans have been diagnosed with TBI, which is considered the signature injury of these wars^1^.

The risk of dementia more than doubles among military Veterans with TBI, but the mechanisms mediating this increased risk remain poorly understood^2^. Neurovascular dysfunction, including blood-brain barrier (BBB) disruption, may be an important initiating mechanism by which mTBI sets in motion pathogenic cascades leading to chronic neurodegenerative pathophysiology^3–19^. BBB disruption has been examined in a number of pre-clinical models of blast mTBI^10,11,20–25^. We and others have shown that blast exposure causes transient BBB disruption^8,10,26^, suggesting that blast also stimulates short-term neurovascular repair processes that interact with more protracted, co-occurring, pathological cascades that undermine BBB integrity. In this regard, blast has been shown to provoke delayed-onset BBB disruption, days after an initial BBB opening was restored to normal^8^. This delayed-onset BBB dysintegrity occurs in specific brain regions and is prominent in the hippocampus and the cerebellum^8,10,27^.

Multiple clinical studies show the cerebellum is vulnerable to the effects of blast mTBI^11,28–32^. Moreover, biophysical simulations suggest a specific vulnerability of the human cerebellum to blast exposure^33,34^. Pre-clinical models of blast-induced mTBI have revealed persistent cerebellar white matter injury^11,35,36^. The cerebellum is classically appreciated for its role in sensorimotor integration, which is affected by blast and other forms of neurotrauma^11,37–41^. However, it is increasingly clear that the cerebellum also plays important roles in modulating higher order cognitive and behavioral functions that are prominently affected in individuals with blast-related mTBI^30,42,43^. The translational significance of the cerebellum, coupled with its vulnerability to blast-induced mTBI, prompted us to focus on better understanding the mechanisms of BBB disruption in the cerebellum.

In this study, we sought to determine whether cerebellar blast-induced BBB disruption is NOS-dependent and to address whether delayed onset BBB disruption and co-occurring leukocyte infiltration are associated with cerebellar pathophysiology and sensorimotor function.

## Results

### Repetitive blast exposure increases BBB permeability to radiolabeled albumin

**Figure 1:**
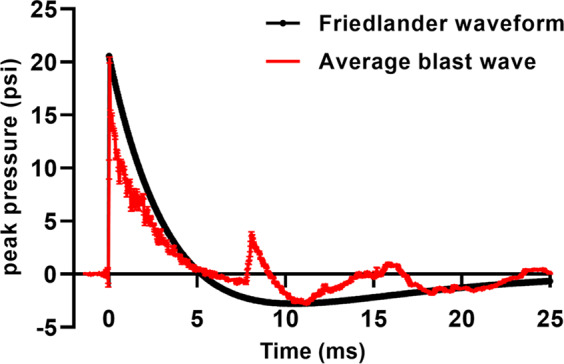
Blast overpressures generated with a well-established pneumatic shock tube simulate a Friedlander waveform expected from high explosives. Red trace denotes the mean waveform of 24 blasts sampled throughout the experiments comprising this report. Black trace shows an estimated Friedlander waveform expected from detonation of approximately 21 kg TNT detonated at a distance of 8 m in the open field. Error bars denote SEM.

Using established methods^8,10,11,14,44–46^, male C57BL/6 mice were exposed to one or three blast overpressures (BOPs) using a pneumatic shock tube delivering a peak static pressure of 20.19 psi (±0.29), positive phase duration of 5.86 ms (±0.065), and impulse of 0.038 psi*ms (±0.0016) (Fig. 1).

**Figure 2:**
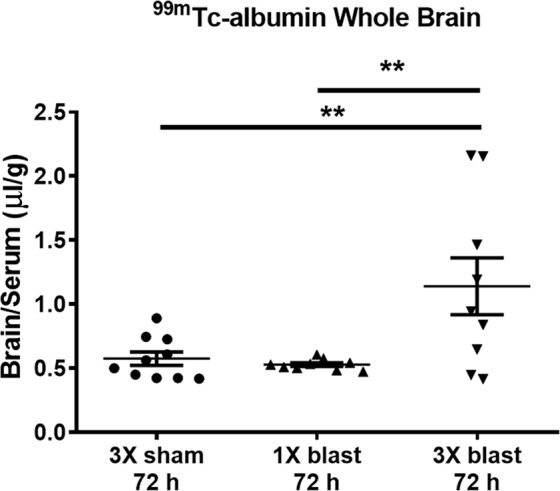
Repetitive blast exposure increases BBB permeability to radiolabeled albumin. A significant increase (**p ≤ 0.01) in brain/serum (uμl/g) ratios of ^99m^Tc-albumin was observed in whole brain at 72 h after repetitive mTBI (3X blast; *n* = 12) compared to 1X blast (*n* = 12), and sham control mice (*n* = 12). One-way ANOVA *post-hoc* Newman-Keuls. Values represent means ± SEM and are expressed as microliters per gram of brain tissue. Brain/serum ratios were calculated by dividing the cpm per brain by the cpm per microliter in the corresponding serum and then by the weight of the brain.

Under normal conditions, only very low levels of blood-borne albumin cross the intact BBB into the CNS^47–49^. We have previously reported that repetitive (2X), but not single (1X) blast causes delayed-onset (72 h) BBB permeability to albumin in whole brain^8^. For the experiments in this report, we used a 3X blast paradigm (one per day for three days) because it better corresponds with commonly used preclinical exposure regimens and is in keeping with our previous translational mTBI findings^11^. The results in Fig. 2 confirmed that in whole brain 3X, but not 1X blast, significantly increased delayed-onset ^99m^Tc-albumin BBB disruption (Fig. 2; *F*_(2,25)_ = 6.94; *p* ≤ 0.01). We have previously shown that this same 3X exposure regimen caused acute BBB disruption, which was closely associated with later emerging chronic neuron loss in the cerebellum^11^. Thus, for this study we focused attention on the effects of 3X blast exposure on BBB disruption in the cerebellum.

### Nitric oxide inhibition blocks albumin permeability in the cerebellum following repetitive blast

**Figure 3:**
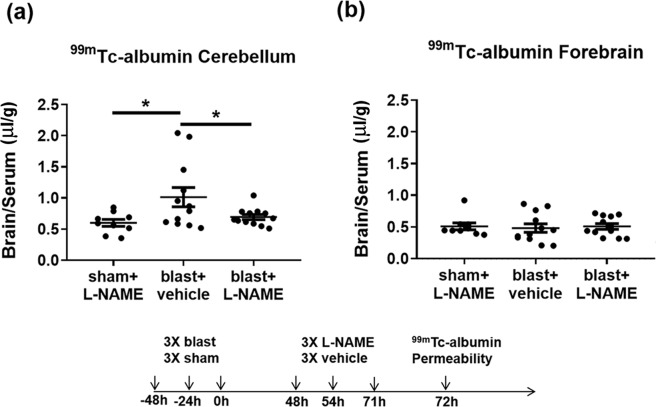
Nitric oxide synthase inhibition blocks blast-induced delayed-onset BBB disruption in cerebellum. **(a)** 3X blast significantly increased brain/serum ratios of ^99m^Tc-albumin in the cerebellum (*p ≤ 0.05), which was significantly attenuated by L-NAME administration (10 mg/kg; ip at 48, 54, and 71 h after the last blast) (*p ≤ 0.05). **(b)** No differences were observed in brain/serum ratios of ^99m^Tc-albumin in the forebrain after repetitive mTBI (*p* > 0.05). One-way ANOVA *post-hoc* Newman-Keuls. Values represent means ± SEM. Timeline portrays the mTBI exposure, L-NAME treatment paradigm, and measurement of BBB permeability.

Nitric oxide (NO) signaling is known to regulate BBB permeability^50–52^. In keeping with this, we have reported that nitric oxide synthase (NOS) inhibition attenuates single and double blast-induced delayed-onset BBB disruption^8^. To address whether 3X blast-induced BBB disruption in the cerebellum is similarly regulated by NOS, we measured uptake of blood-borne ^99m^Tc-albumin 72 h after the final exposure in mice injected with the pan-specific NOS inhibitor, N(G)-nitro-L-arginine methyl ester (L-NAME). We found that 3X blast significantly increased delayed-onset BBB disruption in cerebellum (Fig. 3a; *F*_(2,30)_ = 4.23; *p* ≤ 0.05), which was significantly ameliorated by L-NAME treatment (Fig. 3a; *p* ≤ 0.05). In contrast to the cerebellum, 3X blast under these specific experimental conditions did not increase BBB permeability to blood-borne radiolabeled albumin in forebrain structures that excluded the hindbrain (i.e., posterior midbrain, pons, medulla, and cerebellum) (Fig. 3b; *F*_(2, 30)_ = 0.073; *n.s*.).

### Nitric oxide synthase inhibition blocks blast-induced CD4^+^ T-cell infiltration in the cerebellum

The results above further support the idea that the cerebellum is particularly vulnerable to blast-induced BBB dysfunction and that delayed-onset BBB disruption is mediated (at least in part) by NOS-dependent signaling cascades. In addition, there is evidence that NOS activity underlies T-cell infiltration into the CNS^53,54^, corresponds with BBB breakdown, and occurs within a temporal window consistent with the delayed-onset BBB disruption we observed following blast^8,55,56^. This prompted us to ask: (i) whether blast increases immune cell infiltration in cerebellum, (ii) whether this occurs in a NOS-dependent fashion, and (iii) if blast-induced immune cell infiltration follows the same brain region-specific pattern (i.e., cerebellum versus forebrain) as BBB disruption.

**Figure 4:**
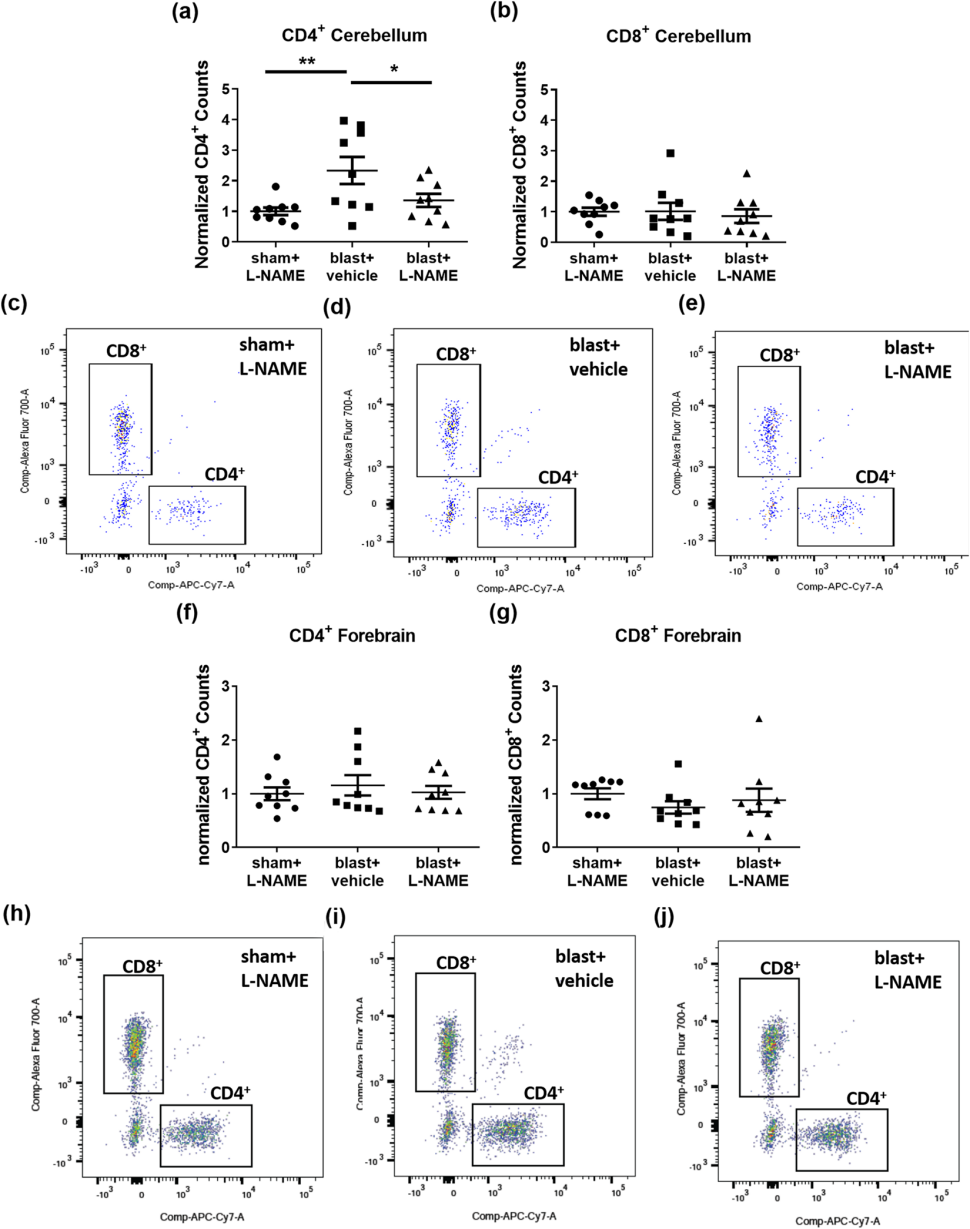
Nitric oxide inhibition attenuates CD4^+^ T-cell infiltration in the cerebellum following repetitive blast. **(a)** Flow cytometry revealed that repetitive TBI significantly increased CD4^+^ counts in the cerebellum (**p ≤ 0.01), which was significantly attenuated by L-NAME administration (*p ≤ 0.05). **(b)** No differences were measured in cerebellar CD8^+^ counts after repetitive TBI (*p* > 0.05). **(c)** Scatter plots for cerebellum of shams, **(d)** blast vehicle-treated, and **(e)** blast L-NAME-treated mice. **(f)** No differences in CD4^+^ counts (*p* > 0.05), or **(g)** CD8^+^ counts were measured in forebrain after repetitive mTBI (*p* > 0.05). **(h)** Scatter *p*lots for forebrain of shams, **(i)** blast + vehicle-treated, and **(j)** blast + L-NAME-treated mice. One-way ANOVA *post-hoc* Newman-Keuls. Values represent means ± SEM.

To test these questions, we employed flow cytometry to quantify CD4^+^ T-cell (CD45^+^/CD3^+^/CD8^−^) and CD8^+^ T-cell (CD45^+^/CD3^+^/CD4^−^) infiltration into cerebellum 72 h after 3X blast exposure (see Methods, Fig. 4, and Supplemental Fig. 1). Blast significantly increased CD4^+^ infiltration into the cerebellum (Fig. 4a; *F*_(2,24)_ = 5.56; *p* ≤ 0.01; scatter plots Fig. 4c-e). Neuman-Keuls post-hoc analyses adjusting for multiple comparisons confirmed that L-NAME treatment blocked blast-induced CD4^+^ T-cell infiltration (blast + vehicle versus blast + L-NAME, *p* ≤ 0.027), reducing infiltration to levels comparable to sham + L-NAME controls. An additional *a priori* planned comparison Helmert analysis further confirmed that CD4^+^ T-cell infiltration into the cerebellum was significantly greater in the blast + vehicle treated group than in the blast + L-NAME and sham control groups (*p* ≤ 0.004); and that the blast + L-NAME group was statistically equivalent to shams (*n.s.*). In contrast to cerebellum, blast exposure did not increase CD4^+^ T-cell infiltration into the forebrain of the same animals (Fig. 4f; *F*_(2,24)_ = 0.331, *n.s.*; scatter plots Fig. 4h-j). Blast exposure did not affect infiltration of CD8^+^ T-cells into either the cerebellum (Fig. 4b; *F*_(2,24)_ = 0.16, *n.s.*) or forebrain (Fig. 4g; *F*_(2,24)_ = 0.69, *n.s.*). We also measured peripheral monocyte (CD11b^+^, CD45 high) infiltration, which was not significantly affected by blast exposure in either the cerebellum (*F*_(2,24)_ = 0.081, *n.s.*) or forebrain (*F*_(2,24)_ = 0.902, *n.s.*).

These results demonstrate that repetitive blast exposure induces brain region-specific, NOS-dependent, CD4^+^ T-cell infiltration that corresponds to the differential effects of blast on BBB integrity in the cerebellum versus forebrain regions. These results, coupled with reports that NOS signaling regulates expression of Intercellular Adhesion Molecule-1 (ICAM-1)^66^, which is known to play a critical role in T-cell transit across the BBB^57,58^, raised the question whether blast increases cerebellar ICAM-1 expression in a NOS-dependent fashion.

### Repetitive blast exposure specifically increases cerebellar ICAM-1, but not VCAM-1 expression in a NOS-dependent fashion

**Figure 5:**
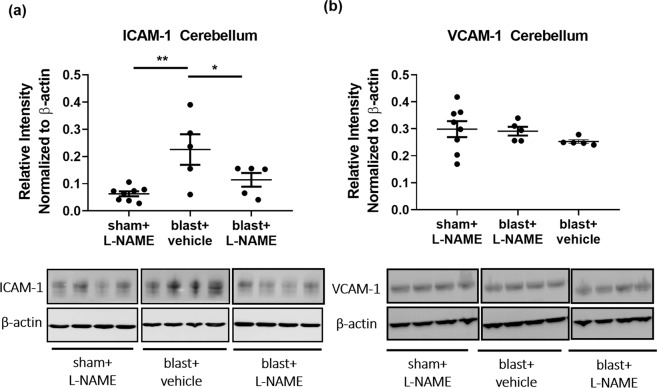
Repetitive blast significantly increased ICAM-1, but not VCAM-1 expression in cerebellum. **(a)** Western blot analysis revealed that repetitive mTBI significantly increased ICAM-1 expression in cerebellum at 72 h (**p ≤ 0.01), and that L-NAME significantly attenuated this effect (*p ≤ 0.05). **(b)** No significant differences were observed in VCAM-1 expression in cerebellum at 72 h after repetitive mTBI (*p* > 0.05). One-way ANOVA *post-hoc* Newman-Keuls. Values represent means ± SEM.

Brain endothelial cell-expressed ICAM-1 plays a critical role in mediating T-cell infiltration into the brain^57^. Thus, we investigated whether blast-induced changes in ICAM-1 could play a role mediating the T-cell results above. Western blot analysis revealed a significant difference in ICAM-1 protein expression in the cerebellum at 72 h after 3X TBI (Fig. 5; *F*_(2,15)_ = 7.66; *p* ≤ 0.01). Moreover, L-NAME administration after blast significantly attenuated ICAM-1 protein expression (Fig. 5a; *p* ≤ 0.05). VCAM-1 is another endothelial membrane protein involved in T-cell tethering^59^. In contrast to the ICAM-1 findings, blast did not alter cerebellar VCAM-1 levels, neither was it affected by L-NAME treatment. We found no significant differences in VCAM-1 protein expression after repetitive blast (Fig. 5b; *F*_(2,15)_ = 0.93; *p* > 0.05).

The increase in ICAM-1 expression corresponds with the observed T-cell infiltration in the cerebellum. Complementing this finding and in keeping with the functional BBB results in Figs. 2 and 3, we found that L-NAME attenuated blast-induced ICAM-1 co-localized with the endothelial cell marker, glucose transporter 1 (GLUT1)^60^ on microvessels in the cerebellum (Supplementary Fig. 4; *F*_(2,9)_ = 10.93; *p* ≤ 0.01). Taken together, these data suggest that repetitive TBI increases cerebellar ICAM-1 expression in a NOS-dependent fashion to regulate endothelial T-cell entry into the CNS.

### L-NAME prevents blast-induced sensorimotor impairment

**Figure 6:**
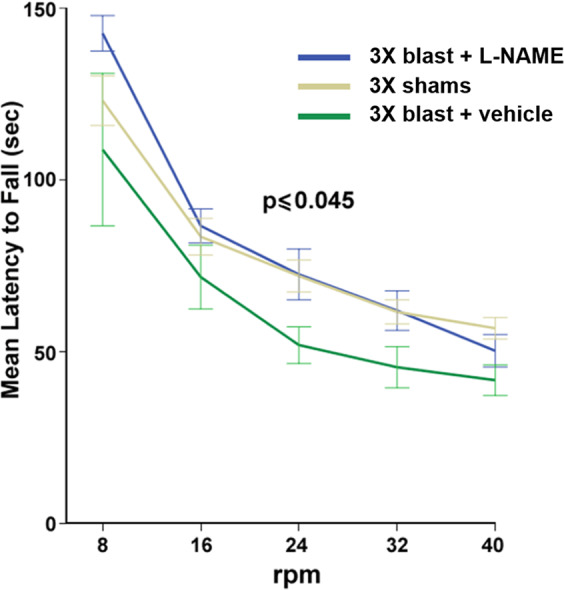
L-NAME improves sensorimotor impairment in mice exposed to repetitive blast. 3X blast and sham mice received L-NAME mice. In shams, L-NAME treatment had no significant effect on performance and were thus pooled (sham-vehicle *n* = 10, and sham L-NAME-treated *n* = 11). There was a significant overall difference (*p* ≤ 0.045) between shams, blast vehicle-treated (*n* = 14), and blast L-NAME-treated (*n* = 14) mice. Mice exposed to repetitive mTBI one month prior exhibited impaired sensorimotor performance on a rotarod task (*p* ≤ 0.05) compared to sham controls (pooled mice, mice administered L-NAME). Post-hoc analysis revealed blast L-NAME-treated mice performed significantly better than blast vehicle-treated mice (*p* ≤ 0.02). Two-way ANOVA Helmert’s test. Values represent means ± SEM.

In cerebella of mice exposed to repetitive TBI, L-NAME prevented BBB disruption (Fig. 3a) and CD4^+^ T-cell infiltration 72 h post-blast (Fig. 4a). To test whether this intervention during the acute/subacute post-blast interval prevents sensorimotor performance deficits, we examined rotarod performance 30 days later. Sensorimotor performance was measured by latency to fall (Fig. 6). Sham and 3X blasted mice received L-NAME or vehicle injections 48, 54, and 71 h after the last blast. We first confirmed that there were no significant effects of L-NAME treatment among sham controls (*F*_(1,19)_ = 3.05, *n.s.*). Thus, vehicle- and L-NAME-treated shams were pooled for comparisons to 3X blast vehicle- and 3X blast L-NAME-treated animals. A two-way mixed (between-within) repeated measures ANOVA revealed a significant overall difference between the sham, blast-vehicle, and blast-L-NAME groups (*F*_(2,34)_ = 3.31; *p* ≤ 0.045). Further post-hoc paired comparison tests confirmed that blast L-NAME-treated mice performed significantly better than blast vehicle-treated (*p* ≤ 0.02) and sham controls (*p* ≤ 0.03). Because L-NAME improved sensorimotor function in mice exposed to TBI, we investigated whether L-NAME treatment influenced the degree of blast-induced neuropathology.

### Repetitive blast causes dystrophic Purkinje cell arbor EAAT4 expression

Using the same blast exposure regimen employed in this report, we have previously found significant Purkinje cell loss occurring in patchy domains consistent with anatomical sub-domains of transient BBB disruption^11^. Following up on this, we examined the effects L-NAME treatment on Purkinje cell arbor structure following 3X blast exposure. To accomplish this, we performed confocal microscopy on cerebella obtained 30 days post-3X blast and immunostained them for EAAT4. EAAT4 is a neuronal glutamate transporter expressed exclusively in the plasma membranes of mature Purkinje cell bodies and their dense dendritic arbors that ramify throughout the cerebellar molecular layer^61,62^.

**Figure 7:**
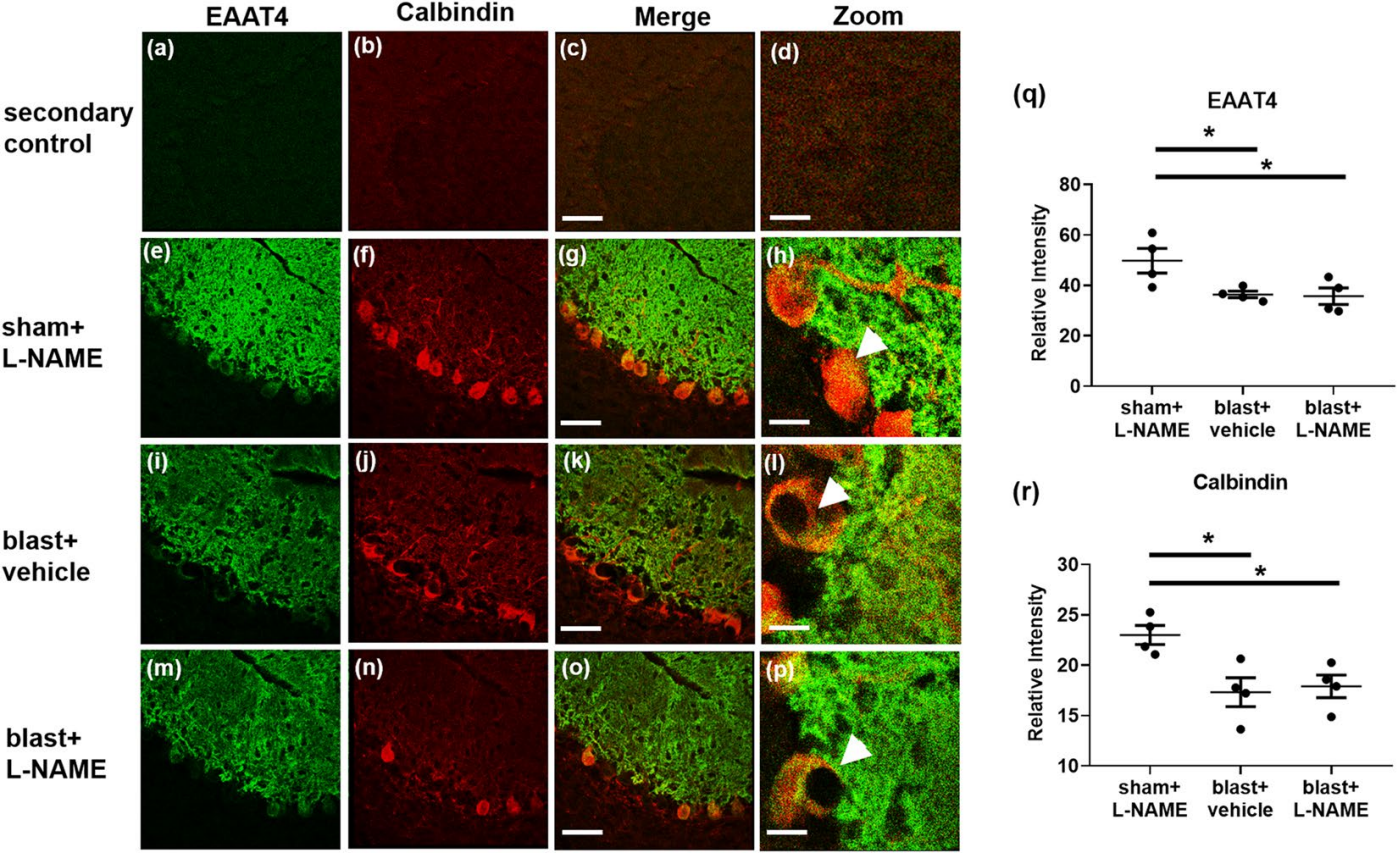
Reduced EAAT4 expression on cerebellar Purkinje cells was observed in mice exposed to repetitive blast injury. **(a)** Shows representative images of secondary only controls (no primary antibodies) for Alexa 488, **(b)** Alexa Cy3, **(c)** merged, and **(d)** zoomed (40×). **(e)** Shows representative immunofluorescent images of EAAT4 (green), **(f)** calbindin (red), **(g)** merged, and **(h)** zoomed images in lobule IX of the cerebellum in control mice at one month after 3X sham and L-NAME administration. **(i)** Shows representative images of EAAT4, **(j)** calbindin, **(k)** merged, and **(l)** zoomed images at one month after repetitive mTBI. **(m)** Shows representative images of EAAT4, (**n**) calbindin, **(o)** merged, and **(p)** zoomed images at one month after repetitive mTBI with L-NAME. A significant decrease in **(q)** EAAT4 (*p ≤ 0.05), and **(r)** calbindin (*p ≤ 0.05) immunofluorescence was observed in lobule IX of the cerebellum of mice exposed to both repetitive mTBI plus vehicle (*p ≤ 0.05), and repetitive mTBI plus L-NAME (*p ≤ 0.05). One-way ANOVA *post hoc* Newman-Keuls. Values represent means ± SEM. Arrowheads highlight EAAT4+/calbindin+ Purkinje cell bodies. Scale bars = 30 µm, 20 µm (zoomed).

Secondary only fluorescent-labeled antibodies were included to validate and support EAAT4 (Fig. 7a), and calbindin (Fig. 7b) morphological specificity (merged Fig. 7c; zoomed Fig. 7d). EAAT4 immunostaining in sham control mice revealed normal-appearing, dense Purkinje cell arbors (Fig. 7e). Normal Purkinje cell morphology was also observed in sham control mice, as evidenced by calbindin immunostaining (Fig. 7f) in lobule IX (merged Fig. 7g; zoomed Fig. 7h). One month after 3X blast exposure, reduced EAAT4 (Fig. 7i) and calbindin (Fig. 7j) immunoreactivity was observed in lobule IX (merged Fig. 7k; zoomed Fig. 7l). This is in keeping with our previous reports of persistent Purkinje cell body pathology, particularly in lobule IX^11^. Of note, L-NAME administration did not rescue the blast-induced decreases in EAAT4 (Fig. 7m) or calbindin immunoreactivity (Fig. 7n) in lobule IX (merged Fig. 7o; zoomed Fig. 7p).

Quantification revealed that repetitive blast mTBI significantly reduced the expression of EAAT4 (Fig. 7q; *F*_(2,9)_ = 5.25; *p* ≤ 0.05), and calbindin (Fig. 7r; *F*_(2,9)_ = 6.93; *p* ≤ 0.05). These results confirm that repetitive TBI causes persistent cerebellar pathology, which may be associated with persistent sensorimotor impairment. Although L-NAME prevented blast-induced sensorimotor deficits, this treatment failed to prevent overt blast-induced cerebellar Purkinje cell neuropathology. This suggests NOS inhibition may possess beneficial effects on persistent functional outcomes after repetitive TBI that are not reflected by gross Purkinje cell loss under these experimental conditions. Nonetheless, this does not rule out the possibility that properly timed NOS inhibition could still be beneficial, or at least delay development of persistent neuropathology in the cerebellum.

### Cerebella from Veterans with blast-related TBI display Purkinje cell dysmorphology

We and others have shown a number of functional and structural neuroimaging abnormalities in Veterans with blast-related mTBI^11,28,32,63–68^. However, currently there is little neuropathological information regarding blast-related mTBI in the cerebellum in humans. In order to gain initial insights into the possible translational significance of the cerebellar pathology in blast exposed mice, we examined cerebella from a rare set of four postmortem brains from US military Servicemembers and Veterans with blast-related mTBI and two comparable non-TBI veteran controls.

Control_1_ was a male Navy officer with no known TBIs who died at 43 due to cardiac arrest (likely myocardial infarction). Medical history included hypertension, hypercholesterolemia, and chronic obstructive pulmonary disease. There were no significant neuropathological abnormalities, including no evidence of tauopathy or neurodegeneration. Control_2_ was a male Army veteran who died at age 57 due to cardiac arrest. He had no lifetime history of TBI. However, he reported falling down the stairs and bumping his head twice at the ages of 4 and 7 years without loss of consciousness or requiring any urgent care or emergency room evaluation. Medical/psychiatric diagnoses included headaches and remote history of a “mental breakdown.” TBI_1_ was a male who died at age 31 of undetermined causes. He had known exposure to high explosives and had a history of PTSD and reported memory and concentration impairment, headaches, anxiety, and depression. Neuropatholgical findings were interface astroglial scarring (IAS), and two tau foci. TBI_2_ was a 46-year-old male Navy SEAL who died from a self-inflicted gunshot wound. Military blast exposure included multiple improvised explosive devices (IEDs) and other explosive ordnance. Contact sports history consisted of high school, college, semi-professional football, and mixed martial arts. Medical/psychiatric history included diagnoses of PTSD, alcohol use disorder, hearing loss, and chronic pain. Neuropathologic findings were CTE (Stage 1) and IAS. TBI_3_ was a male Navy SEAL who died at age 35 by drowning. Military blast exposure included multiple IEDs and other explosive ordnance. There was no history of participation in contact sports. Medical/psychiatric diagnoses included PTSD, alcohol/substance use disorder, chronic pain, paranoid ideation, and manic episodes. Neuropathological findings were IAS without tau pathology. TBI_4_ was a male Army veteran who died at age 46 following complications of elective back surgery. This case had previously participated in a study of blast mTBI and had extensive pre-mortem clinical characterization. Military blast exposure consisted of >50 blast mTBIs due to IEDs and other explosive ordnance with acute symptoms consistent with VA/DoD criteria for mTBI and no history of contact sports or non-blast TBIs. Medical/psychiatric diagnoses included PTSD, alcohol use disorder, migraine headaches, chronic back pain, hearing loss, and tinnitus. Neuropathologic diagnoses included CTE (Stage 1) and meningitis.

**Figure 8:**
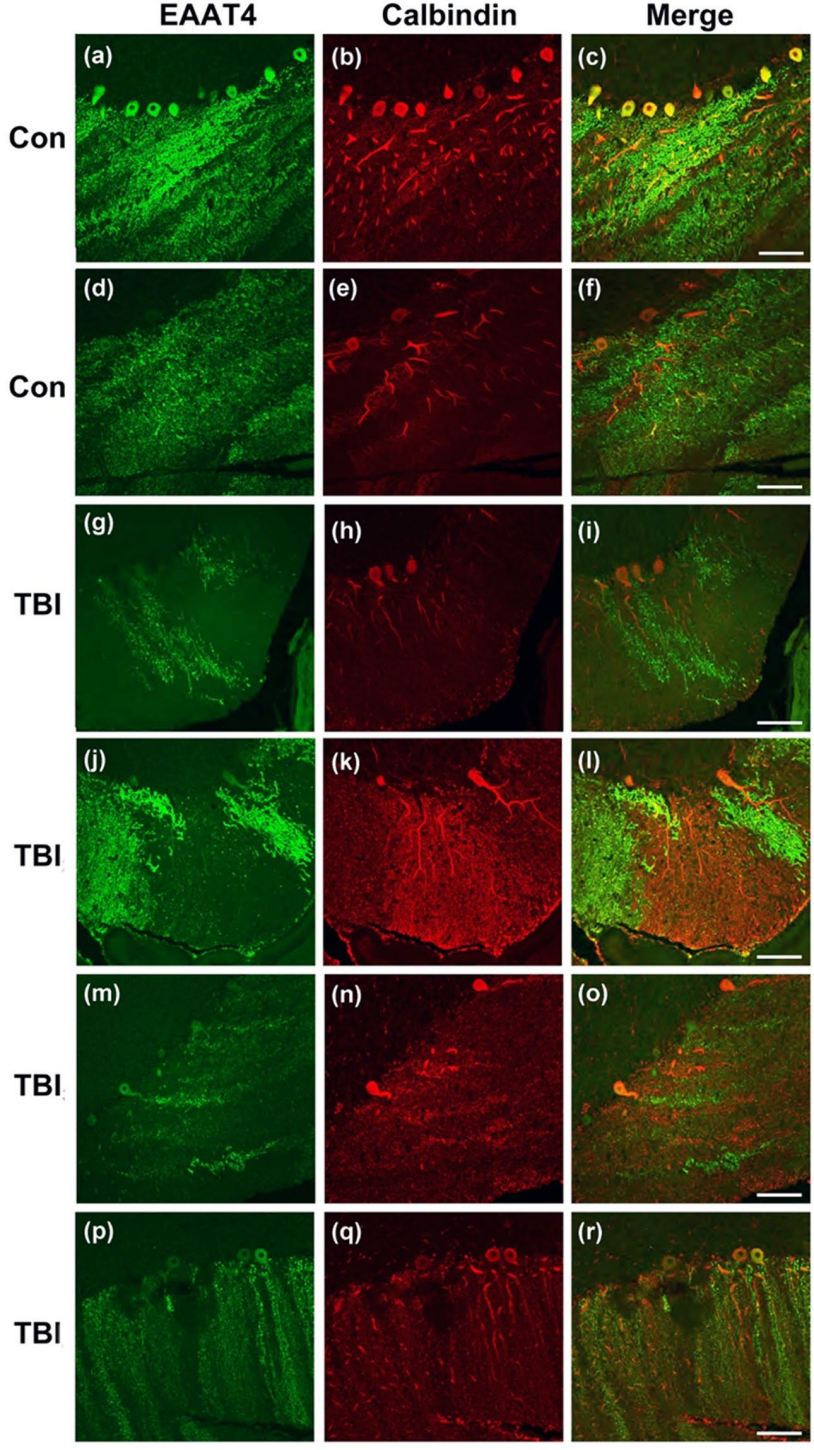
Dystrophic EAAT4 expression in cerebellar Purkinje cells in human with blast-related mTBI. Confocal microscopy shows representative immunofluorescent images of EAAT4 (green), and calbindin (red) in the cerebella of Veteran/military Servicemember controls with no blast TBI (**a**–**c** and **d**–**f**, Controls_1_–_2_, respectively described in Results), and in the cerebellum of Veteran/military Servicemembers with blast-related mTBI (**g**–**i, j**–**l, m**–**o**, and **p**–**r**, TBIs_1–4_, respectively as described in Results) Scale bars = 50 µm.

In keeping with the mouse neuropathologic findings (Fig. 8) and our previous results in 3X blast exposed mice also examined 30 days post treatment^11^, we observed patchy domains of cerebellar Purkinje cell loss (Fig. 8) in the cases with blast-related mTBI (red calbindin staining). Accompanying this, we observed striking patchy domains of EAAT4^+^ arbor loss in the molecular layer (green). In some instances, these arbors were not lost (evidenced by the calbindin immunostaining), yet still displayed a marked decrease in EAAT4, a molecule that is critical in regulating glutamate uptake and glutamatergic signaling in these cells. Overall, these results support the translational significance of the neuropathology we and others have reported in blast-exposed animals^11,14,69,70^, and suggest that the vast network of highly arborized Purkinje cells, which are the sole output of the cerebellum, may be chronically disrupted by blast-related mTBI.

## Discussion

We have found that repetitive mTBI causes delayed-onset BBB disruption in the cerebellum and was markedly more robust than in forebrain structures of blast-exposed mice. Biphasic BBB disruption has been observed in other rodent models of neurotrauma^71,72^. This dynamic property of BBB injury is potentially important because latent BBB disruption has been proposed as a target for post-injury TBI intervention^8^. In this report, we found that the cerebellum was more susceptible to BBB dysfunction than the forebrain. This finding is well in keeping with our previous results showing that the cerebellum is vulnerable to blast-induced BBB disruption^8^, subsequent Purkinje cell loss^11^, and is also in keeping with pioneering findings in other neurotrauma animal models that show cerebellum is prone to injury even when the initiating insult is focused elsewhere in the brain^40,41^.

Previously it has been thought that the CNS and the peripheral immune system are functionally independent of one another. This view has more recently undergone important revisions based on studies that demonstrated active interactions between immune cells in the periphery and in the CNS^73^. In this study, CD4^+^ T-cell infiltration was observed in the cerebellum. Leukocyte infiltration has been considered a functional surrogate for BBB dysfunction, which has been observed in the injured CNS after more severe impact TBI^74^. Recently there has been a report that blast can induce CCL2^+^ monocytes to cross the BBB^9^. The results herein demonstrate that CD4^+^ T-cells infiltrate the cerebellum following repetitive blast-induced mTBI.

A growing body of evidence indicates that neurotrauma can provoke peripheral immune cells to infiltrate the brain^56^. While there is gross histological evidence indicating that leukocytes enter the CNS after blast exposure^75,76^, the effects of blast on immune cell brain infiltration have thus far received limited attention and there has been no information regarding potential mechanisms or functional consequences. One mechanism by which leukocytes and, in particular, T-cells infiltrate the brain involve changes in ICAM-1 expression^57^. We found that ICAM-1 expression was both increased in cerebellum after blast mTBI and was reduced by NOS inhibition. Increased expression of ICAM-1 has also been observed in human endothelial cells exposed to inflammatory cytokines^77^. In addition, NOS signaling modulates ICAM-1 expression and function^53^. Excessive inflammation can cause microvascular hyper-permeability through NOS-dependent mechanisms^78^. Interestingly however, inhibition of constitutive NOS signaling promotes leukocyte adhesion^79^. Blast exposed mice exhibit excessive NOS signaling^80^ and NOS-dependent BBB dysfunction^8^. The data in this report shed new light on the NOS-dependent mechanisms of delayed leukocyte infiltration associated with BBB dysfunction.

In keeping with our previous work on delayed-onset BBB disruption^8^, these results speak further to the potential significance of the acute/subacute post-injury interval in shaping long term outcomes. Even though L-NAME and it’s active NOS inhibiting metabolite L-NG-nitro arginine (LNOARG) have short half-lives on the order of 20 minutes to 22 hours, respectively^81^, we found that L-NAME treatment from 48–71 hours post-blast prevented impaired sensorimotor performance tested 30 days after the last blast exposure.

Changes in NOS signaling have been observed in other pre-clinical brain injury models^82,83^ and have been implicated in disease progression after TBI^84,85^. A current clinical trial using inhaled nitric oxide is underway as a means to improve TBI outcome (NCT03260569 clinicaltrails.gov). The potential relationships between peripheral NO administration, the timing of such treatment post injury, and other factors make it very difficult to draw precise correspondences between the findings in this report, Nonetheless, this effort in the clinical arena lends additional support to the idea NO/NOS signaling pathways are relevant targets for further investigation in treating mTBI. NOS inhibition has also been shown to improve sensorimotor function^38^ and reduce neuronal cell death^86^ in preclinical models of TBI. Our results suggest that acute/subacute NOS inhibition does not block the full spectrum of cerebellar neuropathology. It is possible that this specific intervention may only delay the sequelae of blast mTBI. Nonetheless, it is possible that long-term strategies to modulate NOS function could offer additional neuroprotection; or at least even further delay onset and progression of mTBI-related neurotrauma.

Currently there is very little information from postmortem human cerebellum after blast exposure, primarily because available tissue from such cases is extremely rare. In the cerebella from four Veterans and military Servicemembers with histories of blast-related TBI, we found marked dystrophic Purkinje cell arbor morphology and loss of EAAT4 expression, which strengthens the potential translational significance of the neuropathologic findings we and others have observed in animal models of blast mTBI.

The vulnerability of the cerebellum to mTBI is not well understood. It is likely due to a number of interacting factors that include: (i) high metabolic demand due to the very large number of neurons (69 billion of the 86 billion total neurons in adult human brain are in cerebellum); (ii) a distinct microvascular structure, and (iii) its anatomical location with respect to the skull^87–89^. In considering these factors, BBB disruption and/or immune cell infiltration may still represent key interacting pathologic processes that drive persistent cerebellar dysfunction and neuropathology.

Veterans with mild TBI exhibit aberrant cerebellar function^30,31,90,91^. BBB dysfunction in the cerebellum could set in motion chronic low-grade inflammation associated with NOS signaling that could eventually result in long-term neurodegeneration. Overall, our results argue that NOS plays an important role in mediating cerebellar responses to mild blast injury and suggest that properly timed interventions blocking NO signaling delay pathophysiological progression. Not only do these complex interactions play important roles in mediating delayed BBB dysfunction, but they may also play a critical role in mTBI neurodegenerative progression.

## Methods

### Animals

Male C57BL/6 (*n* = 148) mice (Jackson Laboratories) 8–12 weeks of age were studied. Mice had *ad libitum* access to food, water, and were group housed. Animals were randomly assigned to sham, drug, or TBI groups. All studies were approved by the Veterans Affairs Puget Sound Health Care System’s Institutional Animal Care and Use Committee. Experiments were conducted in accordance with the National Institutes of Health Guide for the Care and Use of Laboratory Animals and reported in compliance with the ARRIVE guidelines.

### Blast exposure

Mice were acclimatized to the animal facility for at least one week prior to experimentation. For blast exposure, animals were first anesthetized with 5% isoflurane, followed by maintenance with 2% isoflurane (1 L/min oxygen). Mice were mounted in a restraint harness in the shock tube with their ventral body surface facing the oncoming shock wave in a pneumatic shock tube as previously described^10,11,44^. Sham control animals were mounted in the restraint harness and held under anesthesia for the same amount of time as the blast-exposed mice. Mice that survived blast exposure (146/148) all displayed normal ambulation, visually guided grasping responses when elevated by the tail near a foothold, and grooming behavior within 1 hour after exposure.

### Radiolabeled tracer preparation

Following established procedures^50^, albumin (Sigma, St. Louis MO) was labeled with ^99m^Tc (GE Healthcare, Piscataway, NJ). A mixture of 240 mg/ml stannous tartrate and 1 mg/ml albumin was adjusted to pH 3.0 with HCl. One millicurie of ^99m^Tc-NaOH_4_ was added to this mixture and allowed to incubate for 20 min. The ^99m^Tc-albumin was purified on a column of G-10 Sephadex (GE Healthcare) in 0.1 ml fractions of phosphate buffer (0.25 M). Radioactivity in the purified ^99m^Tc-albumin peak was more than 90% acid precipitable in an equal volume of 1% bovine serum albumin (BSA) and trichloroacetic acid (30%). 5 × 10^6^ cpm/mouse of purified ^99m^Tc-albumin fraction was prepared in a final volume (0.2 ml/mouse) of lactated Ringer’s solution containing 1% BSA.

### Radiolabeled tracer injections

Following established procedures^50^, at 72 h after the final blast/sham treatment, mice were anesthetized with urethane (4 g/kg; 0.2 ml; ip); jugular veins were exposed, and injected with ^99m^Tc-albumin (5 × 10^6^ Counts per minute (cpm)) in 0.2 ml of lactated Ringer’s solution with 1% BSA for 10 min. Blood was collected from a cut in the descending abdominal aorta. Vascular space of the brain was then washed free of blood by opening the thorax, clamping the descending thoracic aorta, severing both jugular veins, and perfusing 20 ml of lactated Ringer’s solution through the left ventricle of the heart in less than 1 min. After washout, brain was removed. The cerebellum and forebrain structures (remaining brain regions excluding posterior midbrain, pons, medulla, and cerebellum) were dissected and individually weighed. Brains with visible blood after washout were excluded from analysis (*n* = 2). Serum was obtained by centrifuging the carotid artery blood for 10 min at 3,200 × *g* at 4 °C. Levels of ^99m^Tc radioactivity in the serum and brain regions were determined in a gamma counter. Brain tissue radioactivity was calculated by dividing the cpm in the brain region by the weight of the brain region to yield cpm/g. Serum radioactivity was calculated by dividing the cpm in the serum by the microliters of serum counted to yield cpm/microliter. The brain tissue radioactivity was then divided by the corresponding serum radioactivity and the results given in units of microliters/gram of brain tissue.

### N(G)-nitro-L-arginine methyl ester treatment

For all studies in this report, L-NAME treatment indicates that the mice received three intraperitoneal injections of N(G)-nitro-L-arginine methyl ester (L-NAME; Sigma; 10 mg/kg; 100 µl 7% NaHCO_3_; ip) at 48, 54, and 71 h after the last blast. For these experiments, shams received identical L-NAME injections along with their respective blast cohorts. We employed this 3X dosing paradigm based on the plasma half-life of L-NAME^81^. Vehicle controls received three 100 µl injections of 7% NaHCO_3_.

### Rotarod

A rotarod apparatus (San Diego Instruments, San Diego, CA) was used to measure motor coordination and balance. Five trials are used, each with a different speed of rotation (8, 16, 24, 32, and 40 rpm). For each trial the rod was accelerated to its final speed over 120 s, remained at the final speed for an additional 30 s, and finally decreased back to 0 rpm over 30 s. 2 min intertrial intervals were used. The latency to fall of the rotarod was recorded for each trial.

### Confocal microscopy

Using previously established methods^8^, brains were post-fixed in 4% paraformaldehyde in PBS at 4° C for ten ^99m^Tc-albumin half-lives (~60 h). Hemisections were equilibrated in 30% sucrose in PBS for 24 h at 4 °C and embedded in OCT (Tissue-Tek, Torrance, CA). Antigen retrieval was performed with 50 mM sodium citrate (pH 9.0) and then heating at 80 °C for 30 min. Immunostaining was performed as previously described^14^. Floating tissue sections were cover slipped with a drop of Prolong Gold Antifade Reagent. The following antibodies were used: rabbit polyclonal anti-EAAT4 (Abcam, Cambridge, MA; 1:1,000), mouse monoclonal calbindin (Abcam; 1:1,000), rabbit polyclonal anti-GLUT1 (Millipore, Billerica, MA; 1:500), and goat polyclonal anti-ICAM-1 (Novus Biologics, Centennial, CO; 1:1,000). Corresponding secondary antibodies labeled with Alexa 488, and Cy3 were applied for 2 h (Jackson Immunoresearch, West Grove, PA; 1:1,000). Confocal microscopy was performed with a Leica TCS SP5 II microscope. Microscopic images were acquired with the Leica Application Suite and processed using image adjustments limited only to linear contrast and brightness adjustments applied identically to data from blast- and sham-treated animals in each experiment.

### Flow cytometry

At 72 h after last blast or after time-matched sham procedures, mice were anesthetized with urethane (4 g/kg; 0.2 ml; ip), and transcardially perfused with ice-cold PBS. Brains were extracted and dissected into cerebellum and forebrain. Brain regions were mashed on ice in PBS. Homogenates were centrifuged at 300 × *g* for 5 min at 4^o^ C. Pellets were resuspended in enzyme dissociation buffer (Tumor Dissociation kit, Miltenyi Biotec, Auburn, CA), triturated with a 22-gauge needle, and rocked at 37 ^o^C. Digested brain suspension were then passed through a 70 μm SmartStrainer and rinsed thoroughly with 5 ml HBSS/FBS/EDTA. The collected suspension was centrifuged at 300 × *g*. Pellets were resuspended in 5 ml of 40% Percoll (GE Healthcare, Pittsburgh, PA, USA) and centrifuged at 400 × *g* for 20 min at 25 ^o^C. The myelin layer (top) and Percoll supernatant were aspirated and the pellets were washed with 5 ml HBSS/FBS/EDTA and centrifuged at 300 × *g* for 10 min at 25 ^o^C. Supernatant was aspirated and pellet was resuspended in 100 μl PBS and transferred to 1.5 ml centrifuge tubes.

Aliquots of each cell suspension were pooled for use as controls for staining. One aliquot from the pool was reserved as an unstained control; a second was heat-treated for 10 min at 55 °C. All cell suspensions, including the pool and the heat-killed aliquot (but not the unstained control) were incubated with an equal volume of viability dye (Ghost Dye510, Tonbo Biosciences, San Diego, CA) diluted 1:500 in PBS for 30 min at 4 °C. Labeling was terminated by the addition of 5 volumes HBSS/FBS/EDTA, and centrifuging at 300 × *g* for 5 min. Cells were resuspended in HBSS/FBS/EDTA containing 5 μg/ml mouse Fc Block (ThermoFisher Grand Island, NY) for 15 min on ice before staining with a panel of fluorophore-conjugated primary antibodies (Supplementary Table 1). Cells were incubated with antibodies on ice for 30 min, covered, then washed and resuspended in 250 μl 1% paraformaldehyde in PBS, and stored at 4 °C until analyzed. Data were acquired on an LSRFortessa SORP using FACSDiva 8.0 (BD Biosciences, San Jose, CA, USA) and analyzed using FlowJo (Tree Star, San Carlos, CA, USA). A standardized gating strategy was used for infiltrating T-cells (Supplementary Fig. 1). Fluorescence-minus-one (FMO) controls were used to determine antibody positivity, and compensation controls were performed by adding 1 drop of UltraComp eBeads (ThermoFisher Invitrogen).

### Immunoblotting

Using previously established methods^8^, brains were flash frozen in liquid nitrogen and stored at −80 °C. Dissected tissue was sonicated in radioimmunoprecipitation assay (RIPA) buffer with protease/phosphatase inhibitors (Thermo; Rockford, IL 1:100), centrifuged for 15 min at 12,000 × *g*, supernatant collected, and protein determined with a bicinchoninic acid (BCA) assay. Protein lysates were resolved by sodium dodecyl sulfate-polyacrylamide gel electrophoresis (SDS-PAGE) (20 µg total protein/lane) under reducing conditions, transferred to nitrocellulose, and probed with goat polyclonal anti-ICAM-1 (Novus Biologics, 1:1,000), rabbit monoclonal anti-VCAM-1 (Abcam, 1:2000), and rabbit monoclonal β-actin (Cell signaling, Danvers, MA; 1:10,000) as loading controls. Blots were probed with anti-goat and anti-rabbit horseradish peroxidase-conjugated secondary antibodies (Jackson ImmunoResearch; 1:5,000) and visualized by enhanced chemiluminescence (GE Healthcare, Piscataway, NJ). Optical densities of the immunoreactive protein bands were quantified using Image-Quant with background subtraction and normalization to loading control (β-actin). Full immunoblots can be found in Supplementary Fig. 2 (ICAM-1), and Supplementary Fig. 3 (VCAM-1).

### Human tissue

Collection of all brain specimens and case characteristics information were conducted in accordance with approved University of Washington and Uniformed Services University Institutional Review Board (IRB) procedures allowing for use of de-identified tissue samples. Tissue samples were obtained from autopsies of blast-exposed Veterans and age, sex-matched, non-exposed controls. Brain sections were stained using a commercially available kit (Opal Manual IHC Kit, Akoya Bioscience). The following antibodies were applied overnight at 4 °C: rabbit polyclonal anti-EAAT4 (Abcam; 1:1,000), and mouse monoclonal calbindin (Millipore; 1:1,000). Heat-mediated antigen retrieval was performed in AR6 buffer. Stained slides were mounted with ProLong Diamond antifade mountant (ThermoFisher). Confocal microscopy was performed as described above.

### Statistical analysis

Statistical analyses used GraphPad Prism 8.0 8 (GraphPad Software, Inc., La Jolla, CA) and SPSS software (IBM Corp, Armonk, NY). Error bars represent standard error of the mean (SEM). One-way analysis of variance (ANOVA) were followed by Newman-Keuls, *post-hoc* tests. Two-way ANOVAs were followed by a Helmert test^92,93^.

### Notification

The opinions expressed herein are those of the authors and are not necessarily representative of those of the Uniformed Services University, the United States Department of Defense or the United States Army, Navy, or Air Force.

## Supplementary information

Supplementary information.

## Data Availability

Authors will provide published data and relevant protocols upon request to the Principal Investigator by phone or email.
