# Supplementary material for: Nitric oxide synthase mediates cerebellar dysfunction in mice exposed to repetitive blast-induced mild traumatic brain injury
Source: Sci Rep. 2020 Jun 10;10:9420. doi: 10.1038/s41598-020-66113-7 (PMC7287110; doi:10.1038/s41598-020-66113-7)
Supplement: Supplementary file 1 — Supplementary information. [file 41598_2020_66113_MOESM1_ESM.pdf]

Nitric oxide synthase mediates cerebellar dysfunction in mice exposed to repetitive blast-induced mild traumatic brain injury

Aric F. Logsdon, Abigail G. Schindler, James S. Meabon, Mayumi Yagi, Melanie Herbert, William A. Banks, Murray A. Raskind, Desiree A. Marshall, C. Dirk Keene, Daniel P. Perl, Elaine R. Peskind, and David G. Cook*

Supplementary Figures

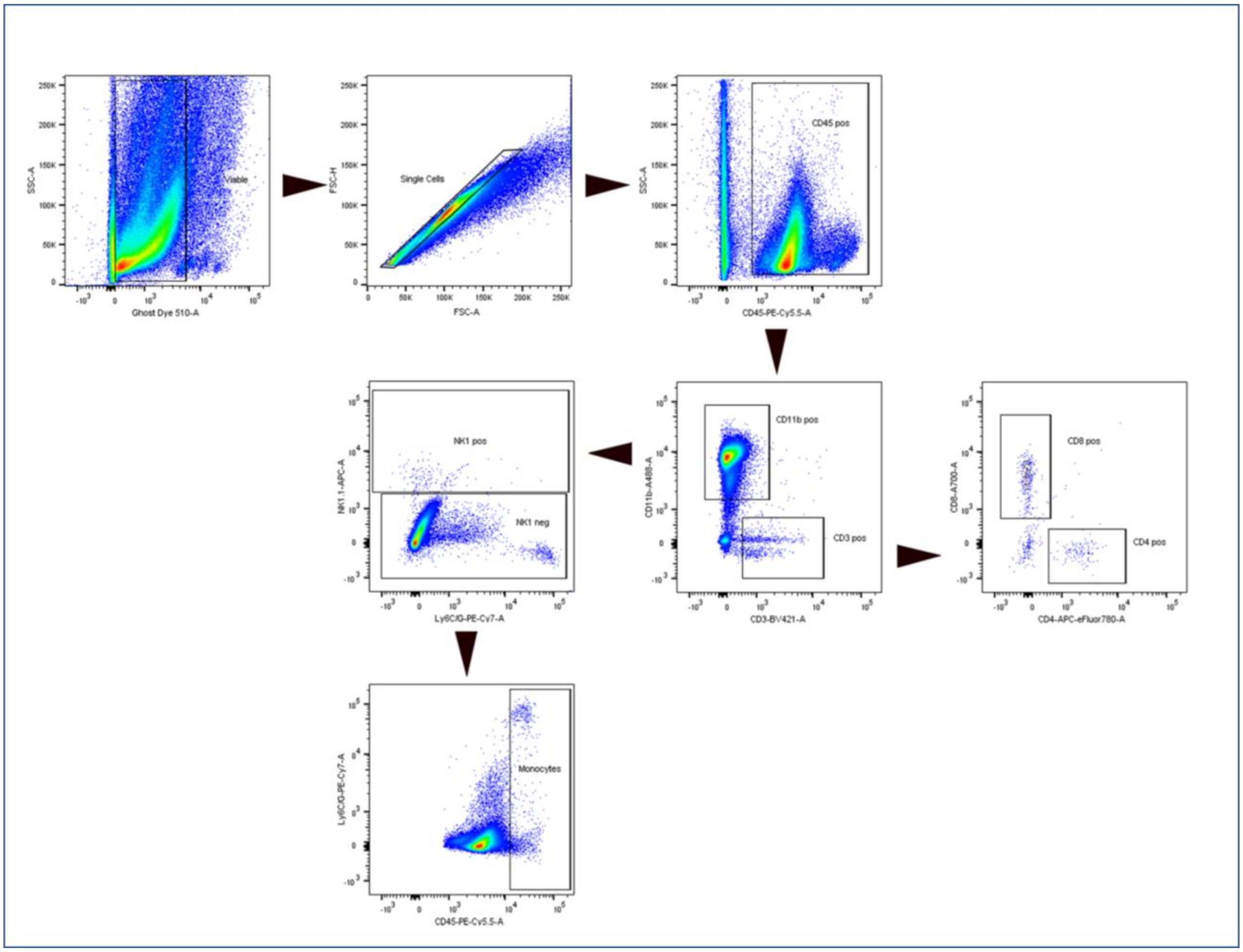

Supplementary Figure 1. Flow cytometry gating strategy. Viable cells were identified and dead cells and debris eliminated by side scatter and low binding of a viability dye. Single cells were identified based on FSC-Area vs FSC-Height. Microglia and leukocytes are characterized by CD45 positivity, and divided by the binding of CD11b (microglia and myeloid cells) or CD3 (T cells). The T cells were classified as CD4+ or CD8+. NK1-negative CD11b-positive cells were identified as microglia (CD45-low) or monocyte/macrophage (CD45-high) based on CD45 expression. FSC-A/SSC-A

analysis of the CD45-high population (not shown) indicated that granulocytes (high SSC-A) were a minority of this population at the time of these analyses.

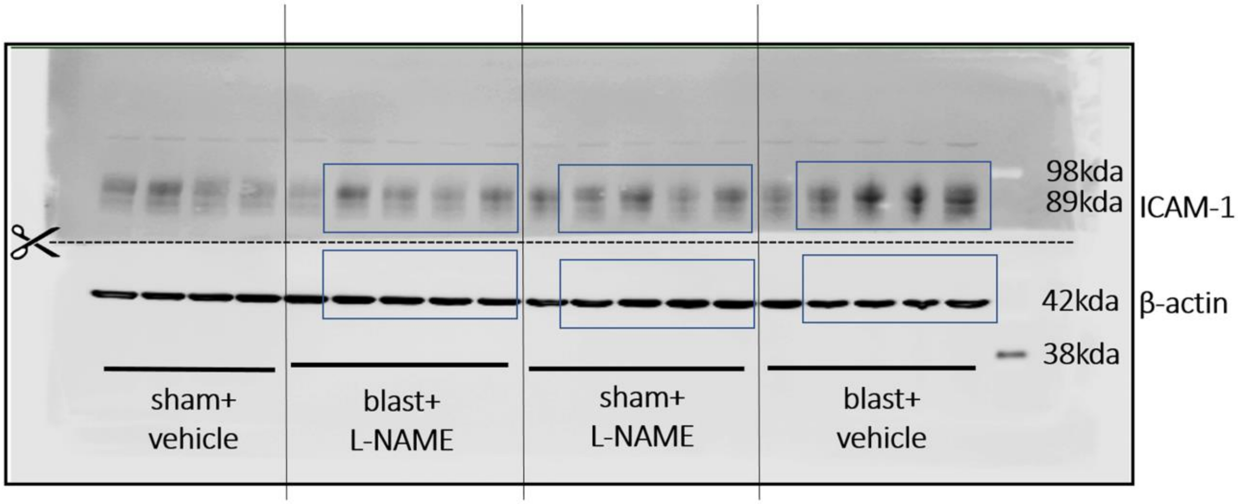

Supplementary Figure 2. Full length immunoblots. Western blot analysis of ICAM-1 (89kDa) and β-Actin (42kDa) from mouse cerebellum. Inset blue boxes demarcate representative immunoblots for ICAM-1 and β-actin in **Figure 5A**.

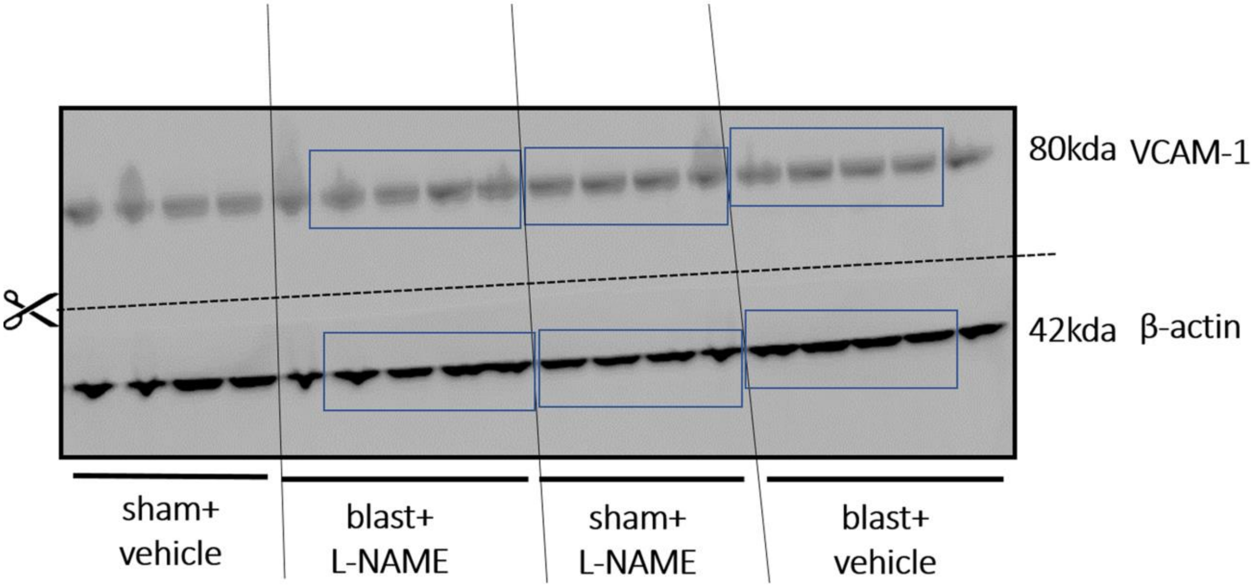

Supplementary Figure 3. Full length immunoblots. Western blot analysis of VCAM-1 (80kDa) and β-Actin (42kDa) from mouse cerebellum. Inset blue boxes demarcate representative immunoblots for VCAM-1 and β-actin in **Figure 5B**.

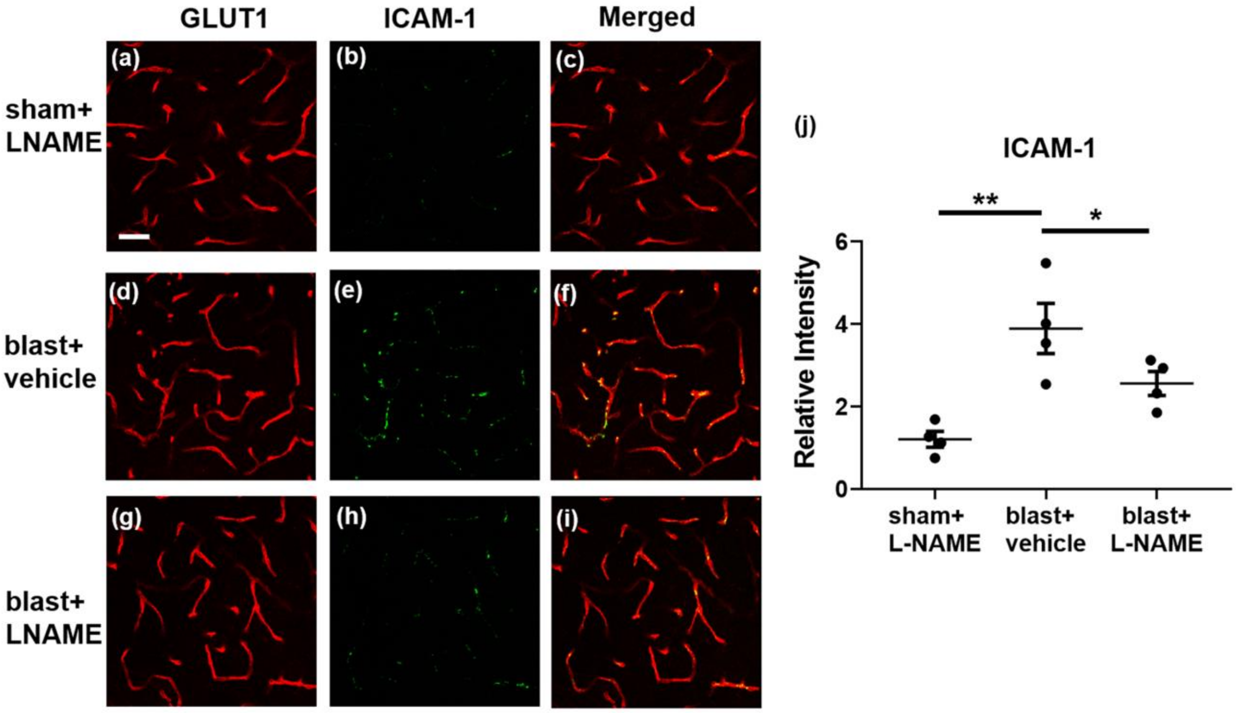

Supplementary Figure 4. Repetitive blast significantly increased ICAM-1 on GLUT1^{+} blood vessels in the cerebellum. **(a)** Shows representative immunofluorescent images of GLUT1 (red), **(b)** ICAM-1 (green), and **(c)** merged within lobule IX of the cerebellum in control mice at 72 h after 3X Sham and L-NAME administration. **(d)** Shows representative images of GLUT1, **(e)** ICAM-1, and **(f)** merged at 72 h after repetitive mTBI. **(g)** Shows representative images of GLUT1, **(h)** ICAM-1, and **(i)** merged at 72 h after repetitive mTBI with L-NAME (10 mg/kg, ip injected 48, 54, and 71 h after the last blast). (**j**) A significant increase in ICAM-1 ($F_{(2,9)}$=10.93; **$p\leq0.01$) was observed in lobule IX of the cerebellum of mice exposed to repetitive mTBI plus vehicle (**$p\leq0.01$), and L-NAME significantly attenuated this effect (*$p\leq0.05$). One-way ANOVA *post hoc* Newman-Keuls. Values represent means±SEM. Scale bars=30 µm.
